# Positive Association of Coronary Calcium Detected by Computed Tomography Coronary Angiography with Periprocedural Myocardial Infarction

**DOI:** 10.1371/journal.pone.0082835

**Published:** 2013-12-17

**Authors:** Xinguo Wang, Xuxia Liu, Hailong Ge, Qing Yang, Xiaoli Liu, Dongmei Shi, Yujie Zhou

**Affiliations:** 1 Department of Cardiology, Beijing Anzhen Hospital affiliated to Capital Medical University, Beijing, China; 2 Beijing Institute of Heart, Lung and Blood Vessel Diseases and The Key Laboratory of Remodeling- related Cardiovascular Disease, Ministry of Education, Beijing, China; University of Groningen, The Netherlands

## Abstract

**Background:**

Periprocedural myocardial infarction (PMI) may occur in approximately 5% to 30% of patients undergoing percutaneous coronary intervention. Whether the morphology of coronary plaque calcium affects the occurrence of PMI is unknown.

**Materials and Methods:**

A total of 616 subjects with stable angina and normal baseline cardiac troponin I levels who had undergone computed tomography angiography (CTA) were referred to elective percutaneous coronary intervention. The morphology of coronary calcium was determined by CTA analysis. PMI was defined as an elevation in 24-h post-procedural cardiac troponin I levels of > 5 times the upper limit of normal with either symptoms of myocardial ischemia, new ischemic electrocardiographic changes, or documented complications during the procedure. Logistic regression was performed to identify the effect of the morphology of coronary calcium on the occurrence of PMI.

**Results:**

According to the presence or morphology of coronary calcium as shown by CTA, 210 subjects were grouped in the heavy calcification group, 258 in the mild calcification group, 40 in the spotty calcification group and 108 in the control group. The dissection rate was significantly higher in the heavy calcification group than in the control group (7.1 % vs. 1.9%, p = 0.03). The occurrence of PMI in the heavy calcification group was significantly higher than that in the control group (OR 4.38, 95% CI 1.80–10.65, p = 0.001). After multivariate adjustment, the risk of PMI still remained significantly higher in the heavy calcification group than in the control group (OR 4.04, 95% CI 1.50–10.89, p = 0.003).

**Conclusions:**

The morphology of coronary calcium determined by CTA may help to predict the subsequent occurrence of PMI. A large amount of coronary calcium may be predictive of PMI.

## Introduction

Periprocedural myocardial infarction (PMI) may occur in approximately 5% to 30% of patients undergoing percutaneous coronary intervention (PCI) [1], [2] and it increases long-term myocardial infarction and mortality [3]–[6]. Furthermore, in a recent study using magnetic resonance imaging (MRI), post-PCI cardiac troponin I level elevation was associated with new, irreversible myocardial injury on delayed-enhancement MRI [7]. A large plaque volume was identified as a risk factor of PMI in an integrated backscatter intravascular ultrasound (IVUS) study [8]. A large necrotic core area in plaques was also identified as a risk factor of PMI in a virtual histology IVUS [9] study. Furthermore, more calcium was observed in lesion sites in subjects with PMI than in those without PMI in an IVUS study [10].

With the temporal and spatial development of multidetector computed tomography (MDCT), it is possible to predictively evaluate the association of coronary plaque characteristics and subsequent PMI in a non-invasive manner. Coronary calcification is associated with spontaneous myocardial infarction and mortality. Nevertheless, it is unknown whether coronary plaque calcification is associated with subsequent PMI. Spotty calcification in coronary culprit lesions is an independent risk factor for developing PMI as shown by MDCT [11]. Circumferential plaque calcification has also been suggested as a risk factor of slow flow during PCI [12]. The volume and fraction of low-attenuation plaques detected by MDCT has been determined to be associated with PMI [13].

We hypothesized that the morphology of overall coronary calcification per patient predictively detected by computed tomography coronary angiography (CTA) is associated with the risk of PMI. The identification of risk factors of PMI before a PCI procedure may help reduce the incidence and extent of PMI and optimize the clinical outcome of PCI.

## Methods

### Population

A total of 1040 subjects with stable angina [14] were invited to participate in the study. The subjects were screened by CTA and subsequently had coronary artery angiography (CAG) performed in Beijing Anzhen Hospital from January 2011 to August 2012. The main exclusion criteria included elevation of baseline cardiac biomarkers (cardiac creatinine kinase MB or cardiac troponin), a previous history of PCI or coronary artery bypass graft surgery, left ventricular dysfunction (ejection fraction <35%), and contraindication to prolonged dual antiplatelet therapy. All subjects gave their written informed consents before participating in the present study. The present study was approved by the Ethics Committee of Beijing Anzhen Hospital and conducted according to the Helsinki Declaration.

### CT angiography protocol

**Figure 1:**
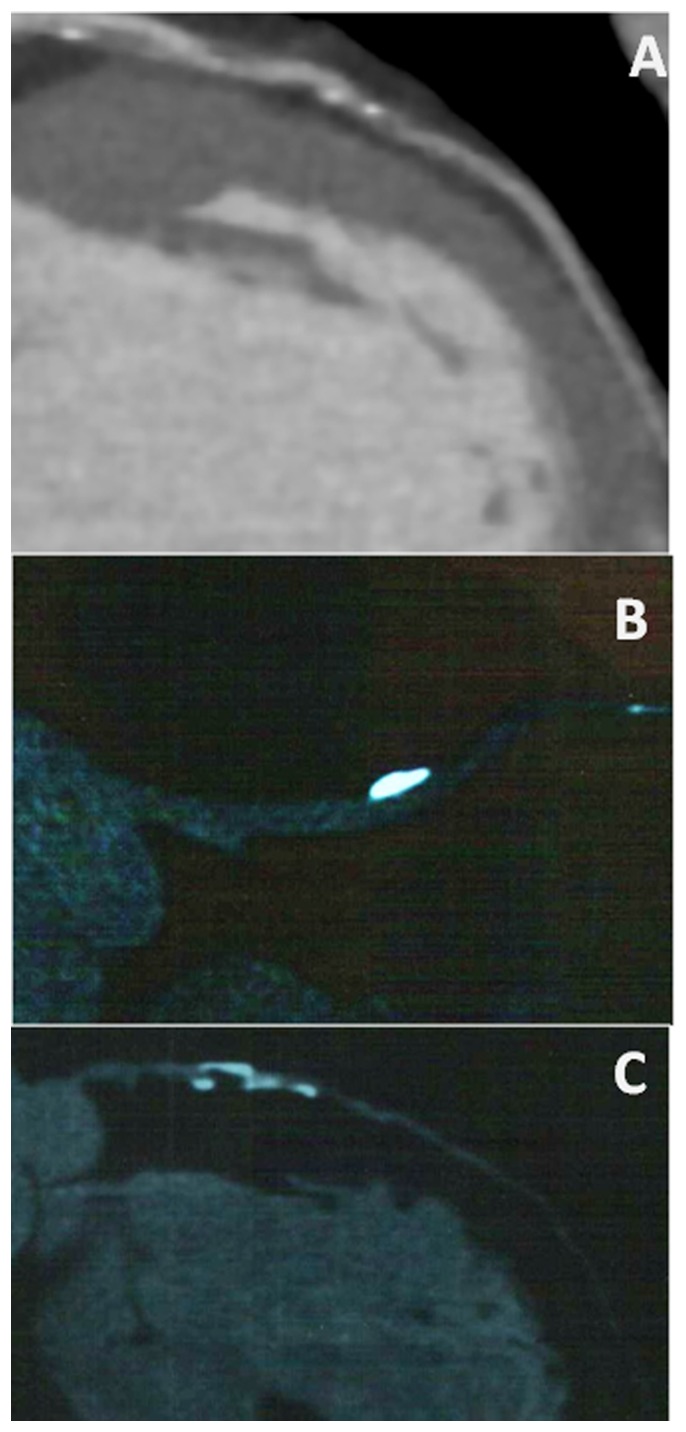
Coronary calcification was grouped as follows: spotty, length of calcium <3/2 of the vessel diameter and width <2/3 of the vessel diameter (A); medium, length of calcium ≥3/2 of the vessel diameter and width <2/3 of the vessel diameter or length of calcium <3/2 the vessel diameter and width ≥2/3 of the vessel diameter (B); and heavy, length of calcium burden ≥3/2 of the vessel diameter and width ≥2/3 of the vessel diameter (C).

Scanning was performed with a dual source 64-CT scanner (Somatom Definition, Siemens Medical Solutions, Forchheim, Germany) according to a previous protocol [15]. Coronary calcium scoring was performed before other CTA analysis [16]. Images were initially reconstructed at the mid-diastolic phase (75% of the R-R interval) of the cardiac cycle. The presence or absence of adherent calcium deposits in or adjacent to each plaque was determined independently by two experienced readers unaware of the patient’s identity, clinical presentation, biomarker analysis, and PCI procedure. If there was disagreement between the two readers, a third reader also evaluated the images. Coronary calcification was classified according to the most severe lesion observed on CTA as heavy, medium (with no areas of heavy calcification), or spotty (with no areas of medium or heavy calcification) (Figure 1) [17].

### PCI procedures

PCI was performed through the radial or femoral artery using a 6-F catheter. During the procedure, anticoagulation was achieved using unfractionated heparin. Use of an adjunctive device or glycoprotein IIb/IIIa receptor was at the physician’s discretion. Once the guide wire passed through the culprit lesion, balloon dilation and stent placement was performed. Angiographic success was defined as angiographic residual stenosis less than 20% of the vessel diameter based on visual estimation and the Thrombolysis In Myocardial Infarction flow was grade 3.

### Biomedical assay

Venous blood samples were acquired on the morning of admission to the hospital, when subjects had been in a fasting state for more than 10 h (baseline), and at 24 h after PCI for determination of serum high sensitive cardiac troponin I (hs-cTnI) levels. Hs-cTnI levels were analyzed with an automated immunoassay (UniCel DxI 800 Access AccuTnI chemiluminescence method, Beckman Coulter, Fullerton, CA, USA). The lower limit in quantification was 0 μg/L and the upper limit of normal in our laboratory was 0.05 μg/L.

### Definition of PMI

PMI was defined as an elevation in hs-cTnI levels of >5 times the upper limit of normal within 24 h post-procedure with either symptoms of myocardial ischemia, new ischemic electrographic changes, or documentd complications during the procedure [2].

### Statistical analyses

Data are expressed as mean ± SD for continuous variables and as percentages for categorical variables. Continuous variables were compared using the analysis of variance test and categorical characteristics by the chi-square or Fisher’s exact test. Inter-reader agreement for calcification category was assessed using the kappa test. Variables with a univariate association with PMI were entered stepwise into a logistic model if their contribution to the model was significant at the α = 0.05 level after mutual adjustment. A binary logistic regression model was applied to adjust for conventional risk factors for PMI and to calculate the odds ratios (ORs). Two logistic models were used to calculate the OR of PMI in the subgroups divided according to coronary calcification. First, age and sex were used as covariate factors for the coronary calcification group for calculation of the adjusted OR. Second, age, sex and other confounding vascular risk factors, including body mass index (BMI), hypercholesterolemia, hypertension, diabetes, current smoking, and diseased vessels, were adjusted in the logistic regression model. In subgroup analysis stratified by elderly, hypertension, diabetes, hypercholesterolemia, and smoking, the trend to PMI across coronary calcification groups was tested within the multivariate models [18]. Logistic regression analysis was also performed according to the category of stented lesion calcification or tertile of coroanry artery calcium score (CACS). A two-tailed P value of 0.05 or less was considered significant. Statistical analyses were carried out using SPSS version 13.0 (SPSS Inc., Chicago, IL, USA.).

## Results

After having undergone CTA, 13 subjects refused to continue the study and were excluded. After CAG, 67 subjects were assigned to have coronary artery bypass graft surgery performed, 344 to medical treatment, and 616 to PCI. According to the presence or morphology of calcification detected by CTA, the population was divided into four groups: 108 without calcification (C group), 40 with spotty calcification (S group), 258 with medium calcification (M group), 210 with heavy calcification (H group). The inter-reader agreement (kappa) for calcification category was 0.99 (p = 0.003).

Subjects with coronary calcification were older than those in the C group (55.1±9.6 years), including those in the S group (63.8±9.7 years, p<0.01, **Table 1**), the M group (60.7±9.8 years, p<0.01), and the H group (62.7±9.4 years, p<0.01). Subjects in the calcification groups were more likely to be hypertensive (H group: 69.5%, M group: 68.2% vs. 55.6%, p<0.05 for both), have a history of hypercholesterolemia (M group: 36.4% vs. 23.1%, p<0.05), and have diabetes mellitus compared with the C group (M group: 31.8%; H group: 37.1% vs. 21.3%, p<0.05, p<0.01, respectively). The CACS was significantly higher in the M group (302.0±121.2) and H group (321.9±115.5) than in the S group (92.0±12.5, p<0.001 for both).

**Table 1:** Baseline characteristics among the four groups of subjects.

Variables	C(n = 108)	S(n = 40)	M(n = 258)	H(n = 210)	P value
Men, % (n)	77.8(84)	75.0(30)	76.7(198)	73.8(155)	0.845
Age, years	55.1±9.6	63.8±9.7^b^	60.7±9.8^d^	62.7±9.4^f^	0.001
BMI, kg/m^2^	26.0±2.60	26.4±3.0	25.9±3.5	26.0±3.0	0.725
% (n)	—	—	—	—	—
Hypertension	55.6(60)	62.5(25)	68.2(176)^c^	69.5(146)^e^	0.011
HCHO	23.1(25)	30.0(12)	36.4(94)^c^	32.4(68)	0.091
DM	21.3(23)	32.5(13)	31.8(82)^c^	37.1(78)^f^	0.035
Smoking	38.0(41)	50.0(20)	40.3(104)	41.9(88)	0.603
History of MI	5.6(6)	5.0(2)	5.4(14)	6.2(13)	0.982
Family of CAD	5.6(6)	5.0(2)	8.1(21)	5.7(12)	0.663
Mean±SD	—	—	—	—	—
TC, mmol/L	4.1±0.8	4.7±1.18	4.38±1.1	4.2±0.9	0.857
HDL-C, mmol/L	1.1±0.2	1.1±0.3	1.1±0.2	1.1±0.3	0.563
LDL-C, mmol/L	2.4±0.6	2.8±0.8	2.6±0.9	2.5±0.7	0.795
TG, mmol/L	1.81±1.14	1.91±1.27	1.75±1.13	1.63±1.05	0.147
FBG, mmol/L	5.8±1.3	6.3±1.5	6.2±1.7	6.0±1.7	0.366
CR, mmol/L	79.3±18.6	82.0±18.7	77.4±16.3	76.3±16.2	0.081
CK, mmol/L	81.2±37.5	98.6±76.6	89.7±48.7	92.7±84.3	0.259
EF, %	65.6±5.7	65.7±7.7	65.3±7.1	64.4±7.3	0.437
CACS	—	92.0±12.5	302.0±121.3	321.9±115.5	<0.001

BMI indicates body mass index; HCHO, hypercholesterolemia; DM, diabetes mellitus; CAD, coronary artery disease; MI, myocardial infarction; TC, total cholesterol; HDL-C, high density lipoprotein cholesterol; TG, triglyceride; FBG, fasting blood glucose; CR, serum creatinine; CK, creatine kinase; LVEF, left ventricle ejection fraction; CACS, coronary artery calcium score.

^b^ p<0.01 between the two groups.^a^ indicates a significant difference of p<0.05 between the S and the C group and

^d^ p<0.01 between the two groups.^c^ indicates a significant difference of p<0.05 between the M and the C group and

^f^ p<0.01 between the two groups.^e^ indicates a significant difference of p<0.05 between the H and the C group and

A significantly higher frequency of triple vessel disease was found in the coronary calcification groups compared with the C group (M group: 24.4%; H group: 35.2% vs. 13.9%, p<0.05, p<0.01 respectively. **Table 2**). The rate of dissection was significantly in the H group than in the C group (7.1% vs. 1.9%, p = 0.03). The diameter of pre-dilation balloons was significantly shorter in the M group (2.2±0.4 mm, p<0.01) and H group (2.1±0.4 mm, p<0.001) than in the C group (2.3±0.3 mm). There was no significant difference in the time of balloon inflation among the four groups. There was a significantly higher numbers of stents (2.1±1.5 vs. 1.6±1.0, p<0.01) and shorter diameter of stents (3.0±0.4 vs. 3.3±0.4 mm, p<0.001) in the H group than in the C group. The stent diameter was also shorter in the S group (3.1±0.4 mm, p = 0.03) and M group (3.1±0.4 mm, p<0.001) than in the C group (3.3±0.4 mm). A total of 1104 plaques were stented, including 389 plaques without calcification (control group), 68 with spotty calcification (SP group), 412 with medium calcification (MP group), and 235 with heavy calcification (HP group).

**Table 2:** Procedural characteristics among the four groups of subjects.

Variables	C(n = 108)	S(n = 40)	M(n = 258)	H(n = 210)	P value
%(n)	—	—	—	—	—
SVD	53.7(58)	42.5(17)	34.5(89)^d^	27.6(58)^f^	<0.001
TVD	13.9(15)	25.0(10)	24.4(63)^c^	35.2(74)^f^	<0.001
LM	6.6(7)	2.5(1)	6.2(16)	10.5(22)	0.161
Ostia	7.4(8)	5.0(2)	4.7(12)	10.0(43)	0.148
Bifurcation	14.8(16)	7.5(3)	11.6(30)	14.3(30)	0.519
CTO	11.1(12)	10.0(4)	11.6(30)	17.1(36)	0.111
Predilation	77.8(84)	67.5(27)	78.7(203)	77.6(163)	0.510
CB	6.5(7)	5.0(2)	6.2(16)	9.0(19)	0.614
Postdilation	64.8(70)	67.5(27)	57.4(148)	55.2(116)	0.238
ST failure	0	5.0(2)	1.2(3)	6.7(14)	0.453
No flow	0.9(1)	0	1.6(4)	1.9(4)	0.637
Perforation	0	0	0.8(2)	0.5(1)	0.611
Dissection	1.9(2)	0	3.1(8)	7.1(15)^e^	0.020
SNC	48.3 (188)	4.6(18)	28.8(112)	18.3(71)	<0.001
SSC	0	72.1(49)	23.5(16)	4.4(3)	<0.001
SMC	0	0	77.2(318)	24.8(94)	<0.001
SHC	0	0	0	100.0(235)	<0.001
Mean±SD	—	—	—	—	—
PP, atm	12.9±3.3	13.6±2.2	13.3±3.1	13.7±3.2	0.302
DP, mm	2.3±0.3	2.3±0.4	2.2±0.4^d^	2.1±0.4^f^	0.001
BDT, s	7.4±1.4	7.2±1.5	7.3±1.4	7.3±1.4	0.969
Number ST	1.6±1.0	1.8±1.1	1.8±1.1	2.1±1.5^f^	<0.001
ST diameter, mm	3.3±0.4	3.1±0.4^a^	3.1±0.4^d^	3.0±0.4^f^	0.018
ST length, mm	23.6±5.0	24.9±5.7	23.8±5.5	24.3±5.2	0.453

SVD indicates single vessel disease; TVD, triple vessel disease; LM, left main trunk artery disease; CTO, chronic total occlusion; CB, cutting balloon; ST, stent; ST failure, stent delivery failure; SNC, stented non-calcified plaque; SSC, stented spotty calcified plaque; SMC, stented medium calcified plaque; SHC, stented heavy calcified plaque; PP, pressure of balloon pre-dilation; DP, diameter of pre-dilation balloon; BDT, time of balloon dilated.

^b^ p<0.01 between the two groups.^a^ indicates a significant difference of p<0.05 between the S group and the C group and

^d^ p<0.01 between the two groups.^c^ indicates a significant difference of p<0.05 between the M group and the C group and

^f^ p<0.01 between the two groups.^e^ indicates a significant difference of p<0.05 between the H and the C group and

The rate of PMI was significantly higher in the H group than in the C group (OR 4.38, 95% confidence interval (CI) 1.80–10.65, p = 0.001, **Table 3**). After adjustment for age, sex, BMI, hypercholesterolemia, diabetes mellitus, hypertension, diseased vessels, and smoking, the risk of PMI was still significantly higher in the H group than in the C group (OR 4.04, 95% CI 1.50–10.89, p = 0.003).

**Table 3:** Logistic regression analysis of the effect of calcification on periprocedural myocardial infarction in the four groups.

Variables	C	S	M	H	P^a^	P^b^	P^c^
Number of subjects	108	40	258	210	—	—	—
Periprocedural MI	6	5	30	43	—	—	—
Crude OR	1	2.43	2.24	4.38	0.163	0.082	0.001
95% CI	—	0.70–8.45	0.90–5.54	1.80–10.65	—	—	—
OR in Model1	1	1.65	1.98	4.59	0.473	0.153	0.001
95% CI	—	0.42–6.48	0.78–5.00	1.82–11.61	—	—	—
OR in Model2	1	1.24	1.93	4.04	0.807	0.218	0.003
95% CI	—	0.23–6.77	0.68–5.52	1.50–10.89	—	—	—

CI indicates confidence interval. Other abbreviations were as followed in Table 2.

Age and gender were adjusted in logistic regression analysis in Model 1; age, sex, BMI, hypercholesterolemia, diabetes mellitus, hypertension, smoking, and diseased vessels were adjusted in Model 2.

P^a^ indicates the significance between the S and the C group in a crude Logistic regression analysis; P^b^ indicates the significance between the M and the C group in a Logistic regression analysis in model 1;

P^c^ indicates the significance between the H and the C group in a Logistic regression analysis in model 2;

To explore the relationship between stented coronary calcification and PMI, logistic regression analysis was also performed. After multivariate adjustment, the risk of PMI was significantly higher in the MP group (OR 2.13, 95% CI 1.30–3.49, p = 0.003, **Table 4**) and HP group (OR 3.43, 95% CI 1.98–5.95, p<0.001), than in the control group. Similarly, the OR of PMI was significantly higher in subjects with a high CACS, in tertile 2 (OR 4.66, 95% CI 1.43–15.20, p = 0.011, **Table 5**) and tertile 3 (OR 3.58, 95% CI 1.12–11.45, p = 0.031) compared with controls without calcification in multivarite adjusted logistic regression analysis.

**Table 4:** Logistic regression analysis of the effect of calcification on periprocedural myocardial infarction according to the morphology of target lesion calcium.

Variables	C	S	M	H	P^a^	P^b^	P^c^
Number of lesions	389	68	412	235	—	—	—
Periprocedural MI	35	6	73	56	—	—	—
Crude OR	1	0.98	2.18	3.16	0.963	<0.001	<0.001
5% CI	—	0.40–2.43	1.42–3.35	2.00–5.01	—	—	—
OR in Model1	1	0.94	2.14	3.40	0.901	0.001	<0.001
95% CI	—	0.38–2.37	1.39–3.30	2.10–5.52	—	—	—
OR in Model2	1	1.20	2.13	3.43	0.715	0.003	<0.001
95% CI	—	0.45–3.23	1.30–3.49	1.98–5.95	—	—	—

Abbreviations were as followed in Table 2.

**Table 5:** Logistic regression analysis of the effect of calcification on periprocedural myocardial infarction according to the tertiles of the coronary artery calcium score.

Variables	C	Tertile1	Tertile2	Tertile3	P^a^	P^b^	P^c^
Number of subjects	108	170	169	169	—	—	—
Periprocedural MI	6	24	27	27	—	—	—
Multivariate OR	1	2.72	4.66	3.58	0.102	0.011	0.031
95% CI	—	0.82–9.01	1.43–15.20	1.12–11.45	—	—	—

Abbreviations were as followed in Table 2.

To further control for the confounding effect of baseline characteristics, subgroup analysis was performed according to the status of elder, diabetes mellitus, hypertension, hypercholesterolemia and smoking. The risk of PMI was significantly higher in the H group than in the C group in subjects with hypercholesterolemia (OR 1.88, 95% CI 1.03–3.43, p = 0.04, **Table 6**). Coronary calcification was demonstrated as the only baseline variable associated with PMI in the final logistic regression analysis (**Table 7**).

**Table 6:** Subgroup analysis of the effect of coronary plaque calcification per patient on periprocedural myocardial infarction in logistic regression analysis.

Variables	Control			Calcification			P for trend
—	n	case	reference	n	case	OR(95% CI)	—
DM	—	—	—	—	—	—	—
yes	23	4	1	173	25	0.95(0.59–1.51)	0.813
no	85	2	1	335	53	1.95(1.31–2.92)	0.001
HTN	—	—	—	—	—	—	—
yes	60	3	1	347	54	1.38(0.95–2.02)	0.095
no	48	3	1	161	24	1.70(1.04–2.78)	0.035
HCHO	—	—	—	—	—	—	—
yes	25	1	1	174	29	1.88 (1.03–3.43)	0.041
no	83	5	1	334	49	1.34 (0.95–1.89)	0.093
Smoking	—	—	—	—	—	—	—
yes	41	2	1	212	27	1.34 (0.85–2.12)	0.214
no	67	4	1	296	51	1.68 (1.13–2.49)	0.010
Elderly	—	—	—	—	—	—	—
yes	22	1	1	204	31	1.11 (0.64–1.90)	0.722
no	86	5	1	304	47	1.67 (1.15–2.42)	0.007

The abbreviations were as followed in Table 2.

Age, sex, BMI, hypercholesterolemia, diabetes, hypertension, smoking status and diseased vessels were adjusted in logistic regression analysis except the subgroup analyzed variable.

**Table 7:** Contribution of baseline variables to PMI in the final logistic regression analysis.

Variables	OR	95%CI	P value
Age, (increment by year)	1.01	0.98–1.03	0.724
Female, (0,1)	1.31	0.70–2.48	0.400
BMI, (kg/m^2^)	1.01	0.93–1.10	0.814
Hypertension, (0,1)	0.82	0.47–1.43	0.478
Diabetes, (0,1)	1.15	0.67–1.98	0.611
HCHO, (0,1)	1.09	0.64–1.86	0.749
Diseased vessel, (0,1,2,3)	1.36	0.97–1.89	0.074
Calcification, (0,1,2,3)	1.55	1.17–2.07	0.010
Smoking, (0,1)	0.91	0.52–1.61	0.753

Abbreviation were as followed in Table 2.

## Discussion

The main finding in the present study was that coronary calcification detected by pre-procedure CTA was significantly associated with PMI (Type 4a MI) according to the universal definition of MI [2]. Heavy coronary calcification was identified as a robust risk factor of PMI after adjustment for baseline characteristics, such as age, sex, BMI, diabetes mellitus, hypertension, hypercholesterolemia, smoking, and diseased vessels.

The significant association of preprocedural coronary calcification and PMI in the present study is consistent with the finding of a previous IVUS study in which more lesion site calcium was observed in subjects with PMI than in those without PMI [10]. Circumferential plaque calcification is associated with a slow flow phenomenon during PCI [12]. However, spotty calcification was found to be an independent risk factor for developing PMI in a MDCT analysis of 107 patients with stable angina [11], which is inconsistent with the finding that heavy calcification may be predictive of PMI in the present study. In another study, the volume and fraction of low-attenuation plaques detected by MDCT of 189 subjects with elective stent implantation were associated with PMI [13]. The small samples size in these two studies may have restricted their statistical significance. Furthermore, the difference in findings between our study and these previous studies may be because of exclusion of severe calcification in the previous studies [11], [13]. In fact, PMI may be considered as a sequence of diffuse atherosclerotic disease [10] and heavy calcification may be associated with the late process of coronary artery disease. Calcium deposition is related to the presence of atherosclerosis and is considered to be direct marker for coronary artery disease, and more severe atherosclerotic plaques tend to have a greater amount of calcium. With the development of calcification, heavy calcification may demonstrate the severity of coronary disease and lead to severe damage of PCI including balloon angioplasty and deployment of stents. Higher SYNTAX scores in CAG analysis are predictive of PMI, while simple lesions (American Heart Association type A and B1) have a high negative predictive value for PMI [19].

The mechanism of PMI after PCI remains unclear, but may be due to occlusion of side branches, coronary spasm, and distal embolization. Coronary dissection may also be one of the causes of PMI, because coronary dissection was significantly higher in the heavy calcification group than in the control group in the present study. The different incidence of PMI among patient groups may also reflect differences in microvascular dysfunction or obstruction of myocardial vessel, which may be caused by periprocedural microembolization of atherothrombotic debris [20]. Coronary calcification may aggravate vascular injury in PCI and subsequently cause damage to cardiac muscle. Calcium phosphate crystals may destabilize atherosclerotic plaques by initiating inflammation and by causing vascular smooth muscle cell death [21]. In fact, larger stent expansion in calcified lesions is associated with a higher incidence of postprocedural MI [22].

In the present study, we found that coronary calcification had a significant effect on PMI in subjects with stable angina pectoris and hypercholesteroemia in subgroup analysis. PMI may be reduced by long-term statin treatment, but not by short-term of high-dose statin treatment [23]. Lipid-lowering therapy has been suggested to have no significant effect on regression of coronary calcification [24]. Coronary calcification may be a marker of late coronary artery disease, and the risk of PMI may be related to this state and predict the severity of coronary artery disease. Physicians should be aware that PCI carries a risk of PMI, especially in patients with heavy coronary calcification.

### Limitation

A limitation of the present study is that the relationship between coronary calcification and the region of PMI was not evaluated. Future research should include evaluation of the region of new myocardial injury using MRI, to evaluate the relationship between coronary calcification and the region of PMI.
